# Supplementary figures and images for: Genetic and Epigenetic Signatures Associated with the Divergence of Aquilegia Species
Source: Genes (Basel). 2022 Apr 28;13(5):793. doi: 10.3390/genes13050793 (PMC9141525; doi:10.3390/genes13050793)

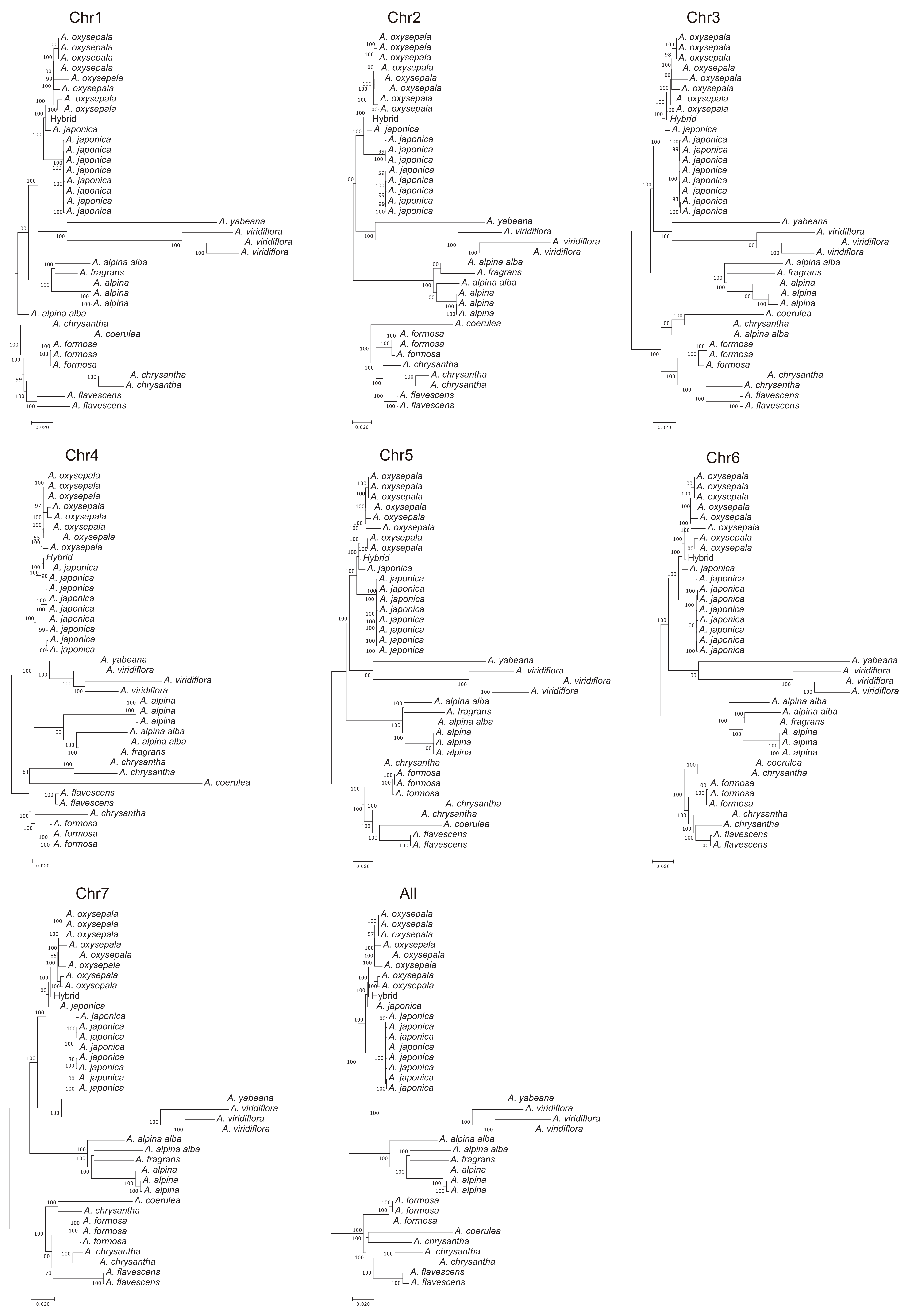

Supplement: Supplementary file 1 [file genes-13-00793-s001.zip › Figure S1.tiff]

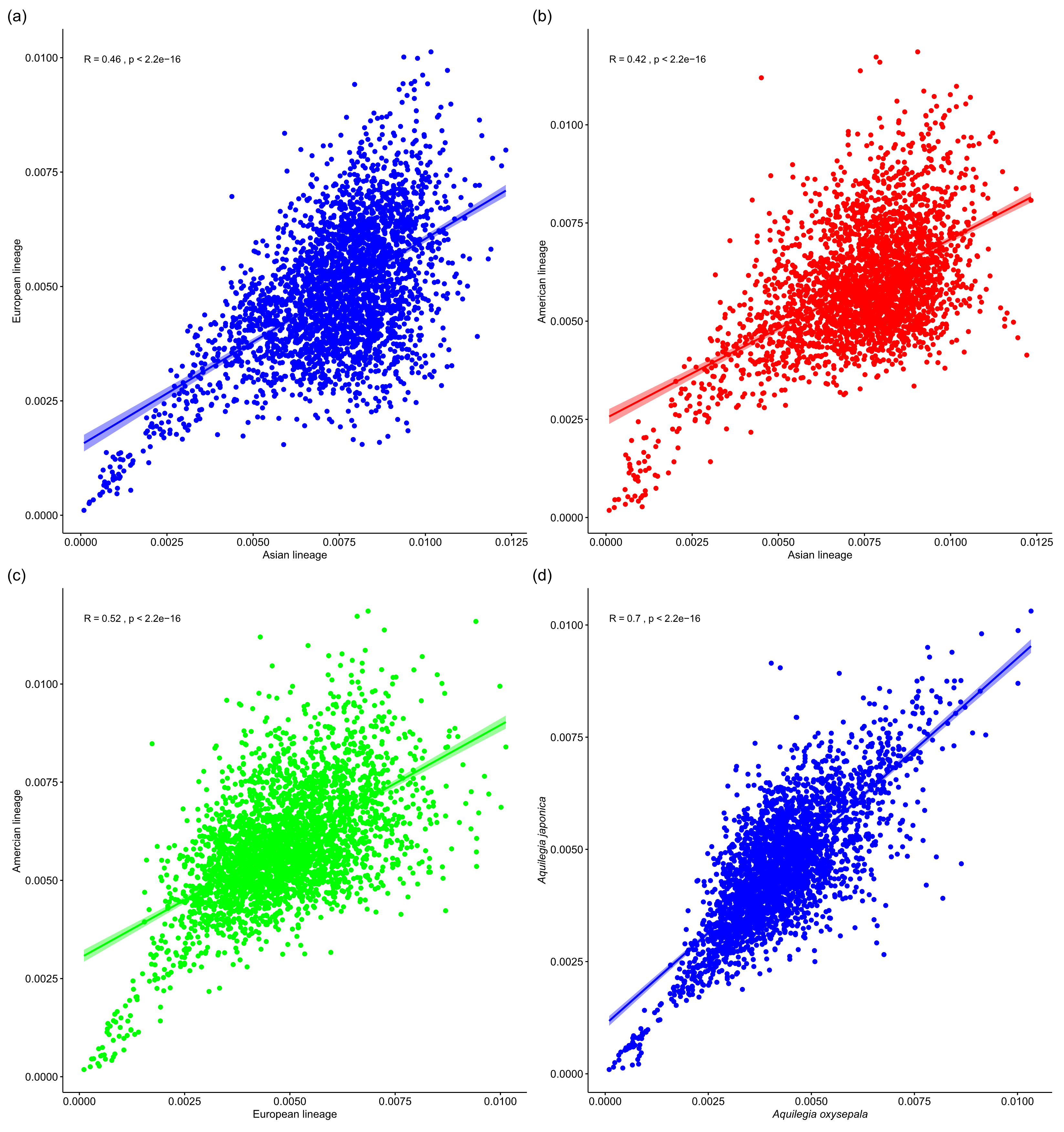

Supplement: Supplementary file 1 [file genes-13-00793-s001.zip › Figure S2.tiff]

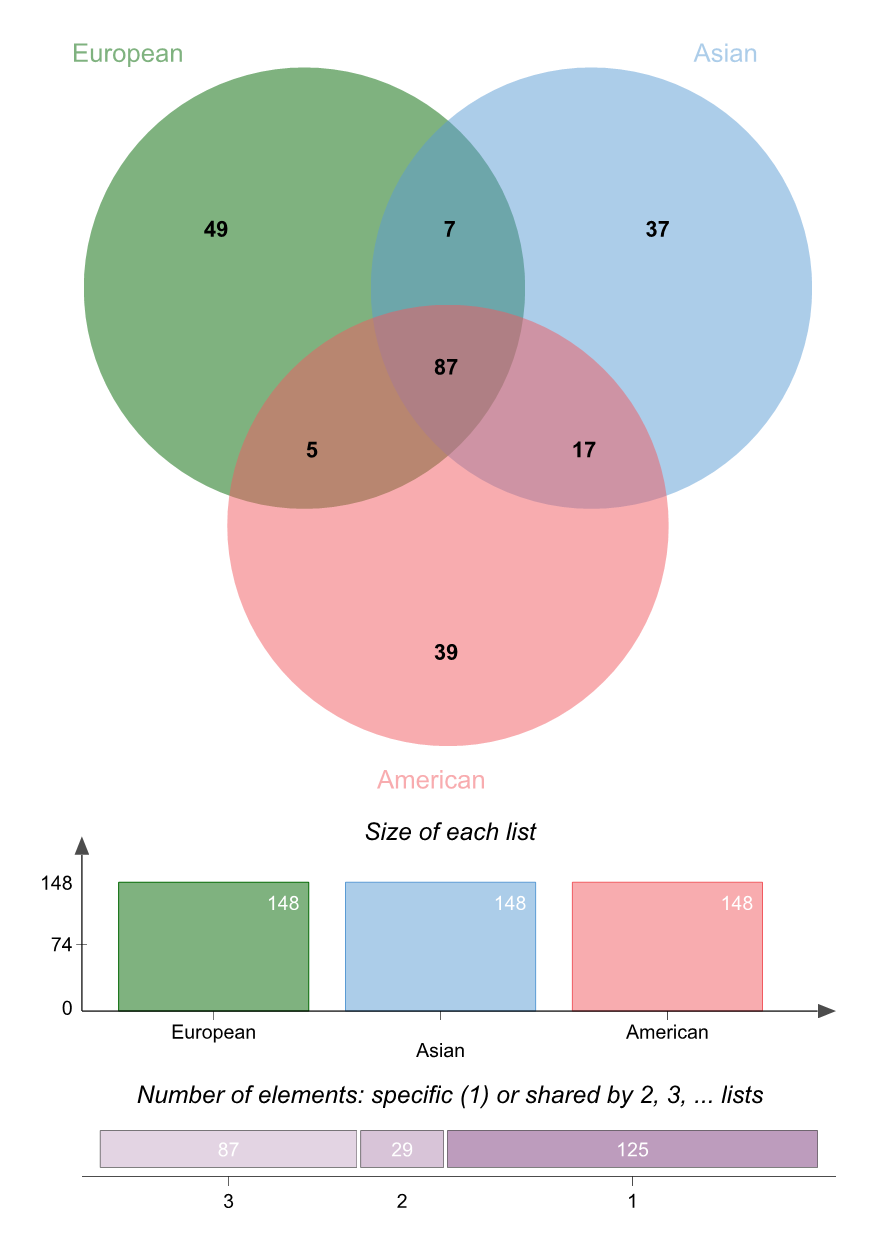

Supplement: Supplementary file 1 [file genes-13-00793-s001.zip › Figure S3.tiff]

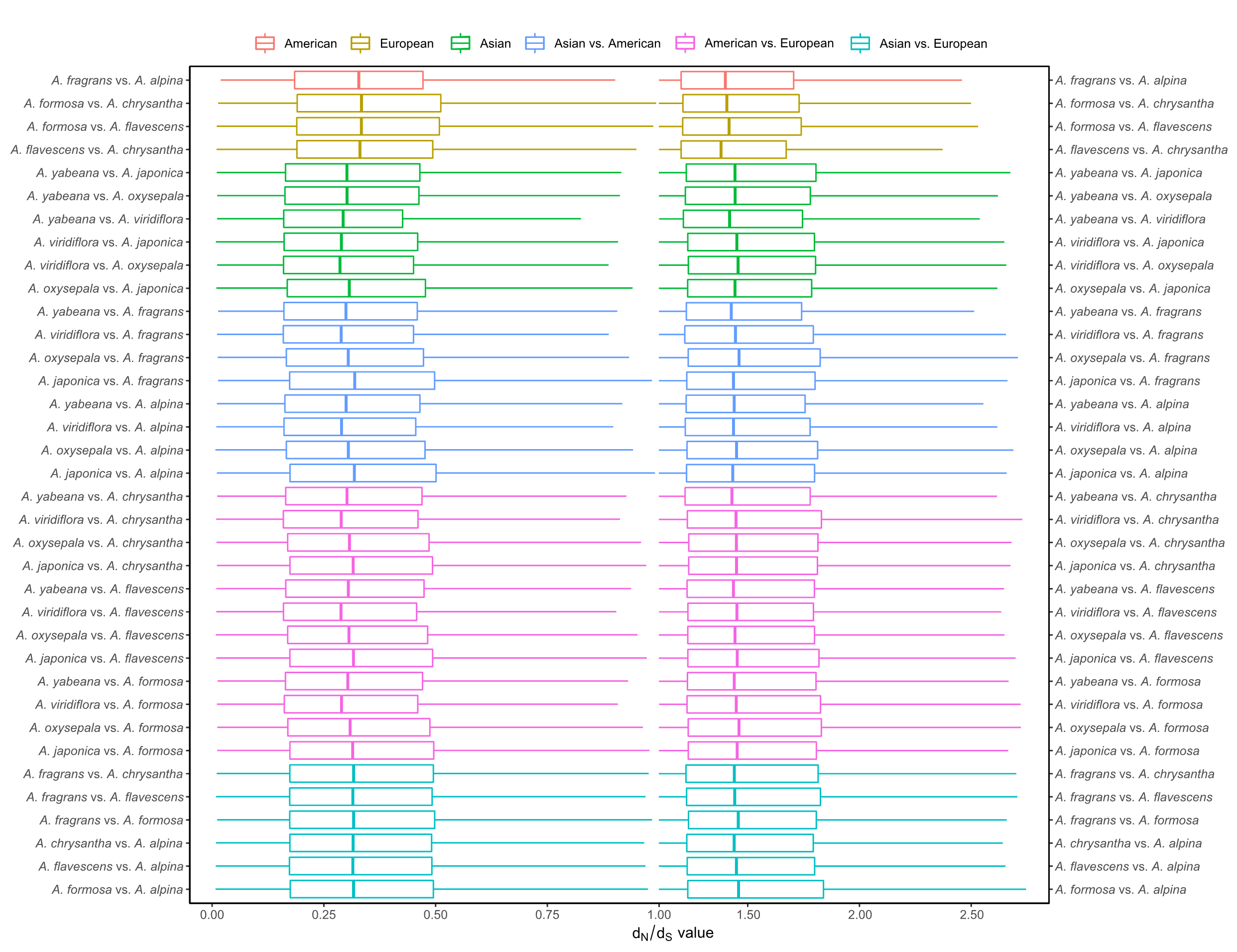

Supplement: Supplementary file 1 [file genes-13-00793-s001.zip › Figure S4.tiff]

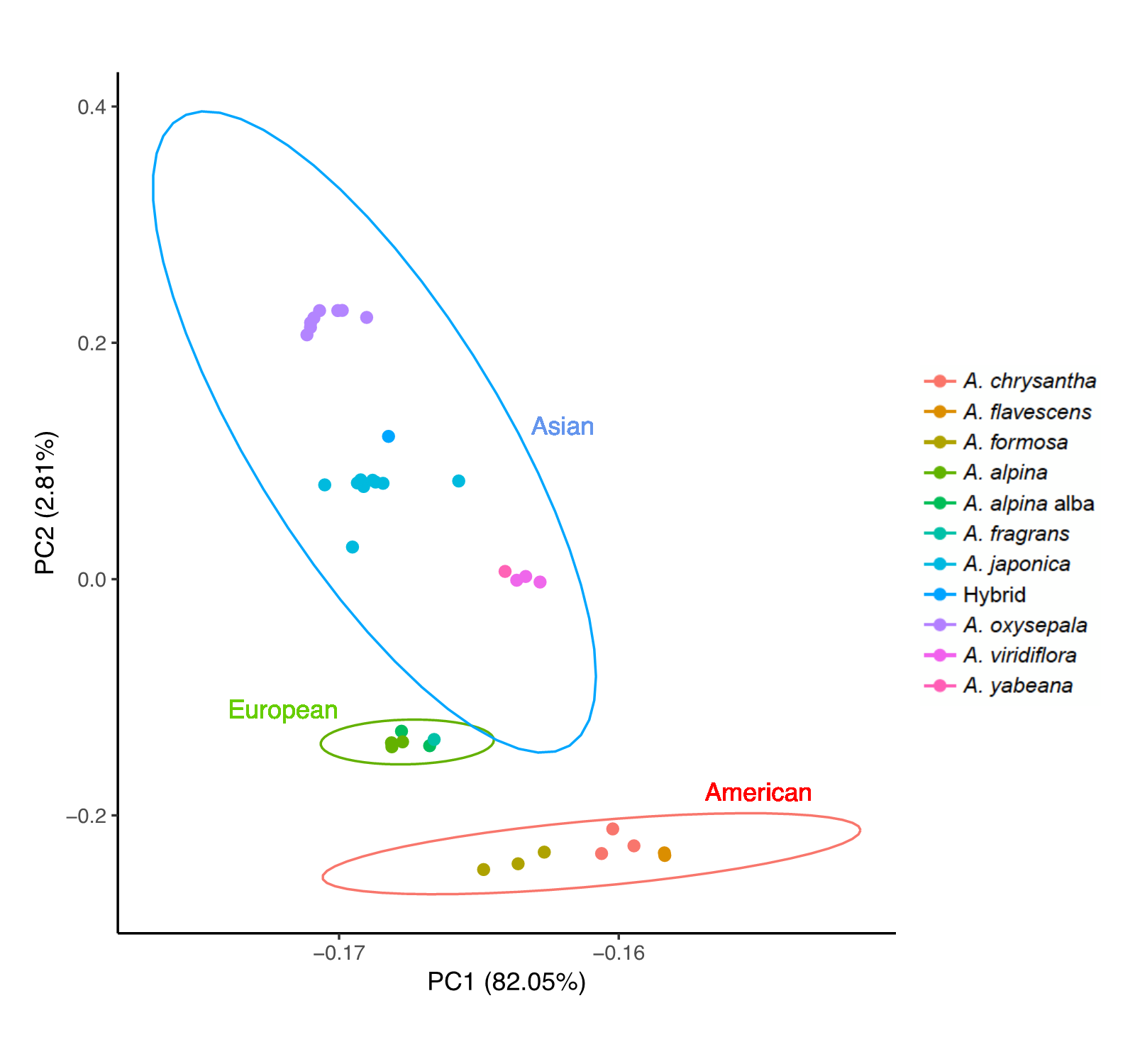

Supplement: Supplementary file 1 [file genes-13-00793-s001.zip › figure S5.tiff]

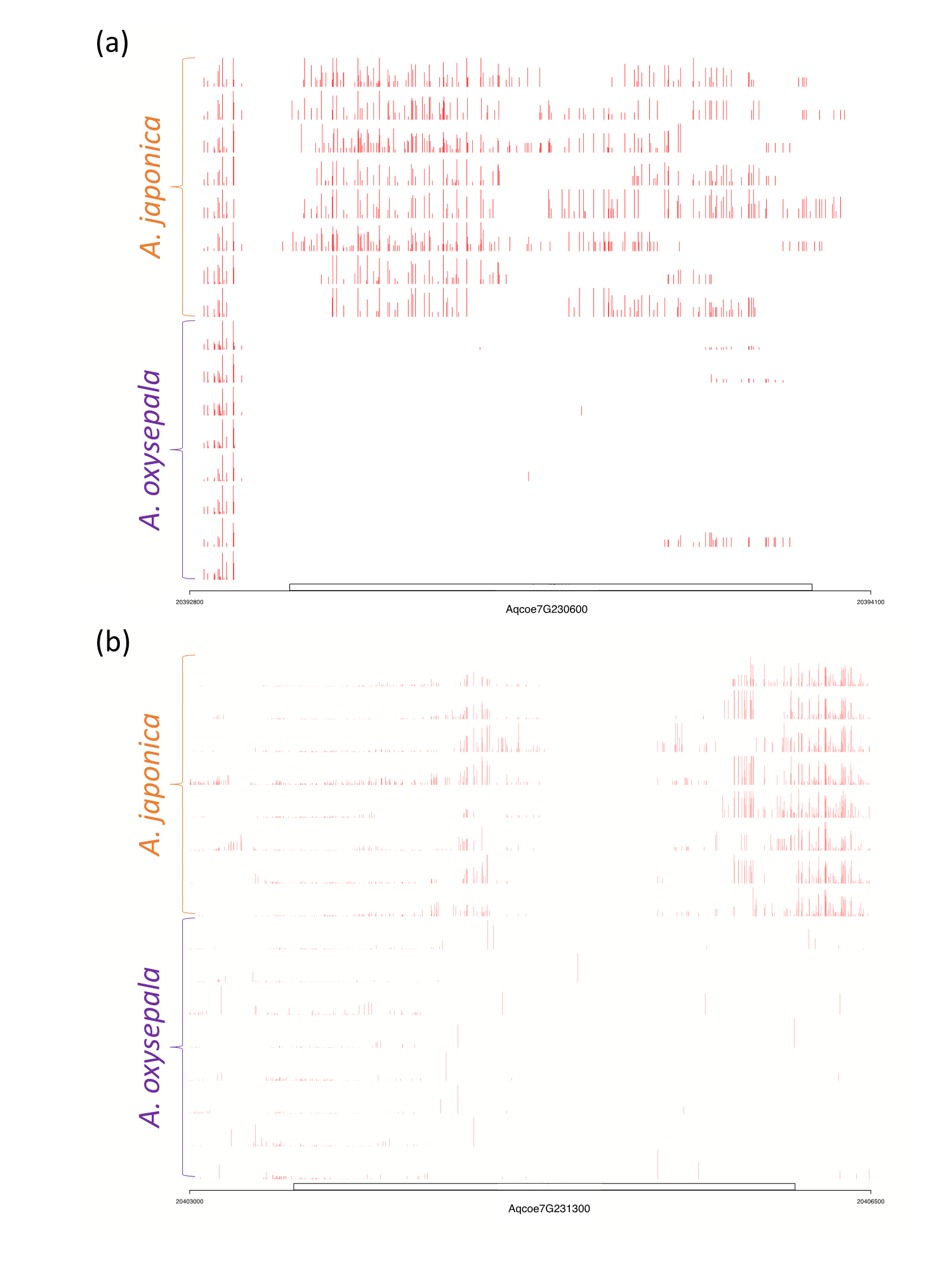

Supplement: Supplementary file 1 [file genes-13-00793-s001.zip › Figure S6.tiff]

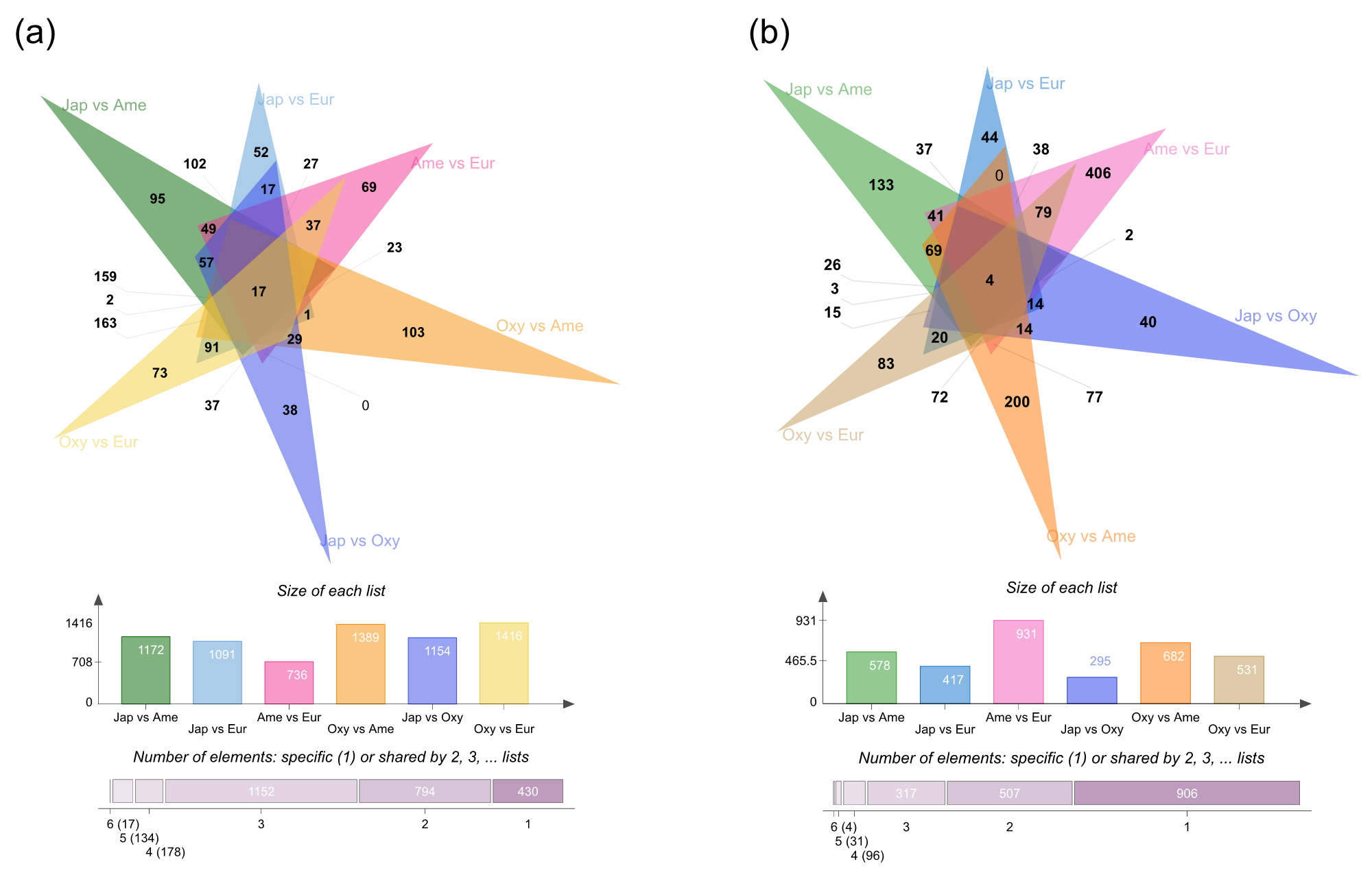

Supplement: Supplementary file 1 [file genes-13-00793-s001.zip › Figure S7.tiff]

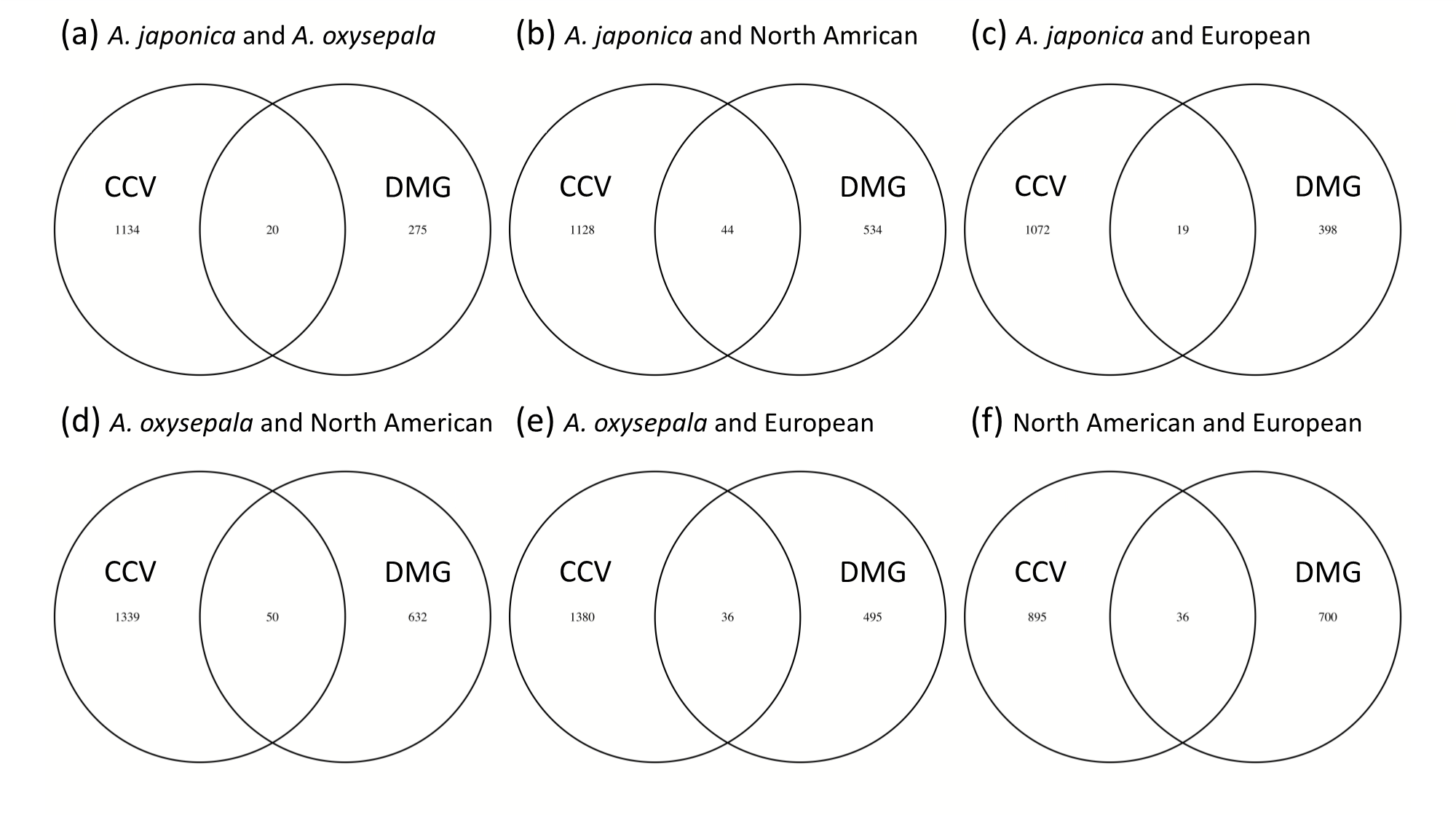

Supplement: Supplementary file 1 [file genes-13-00793-s001.zip › Figure S8.tiff]
